# Editorial: Treatment of Alzheimer’s disease-discovery of natural products based on neurite outgrowth and neuroprotection

**DOI:** 10.3389/fphar.2022.1079783

**Published:** 2022-11-18

**Authors:** Zhiyou Yang, Cai Song, Yue-Wei Ge, Chihiro Tohda

**Affiliations:** ^1^ Guangdong Provincial Key Laboratory of Aquatic Product Processing and Safety, College of Food Science and Technology, Institute of Nutrition and Marine Drugs, Guangdong Ocean University, Zhanjiang, China; ^2^ Key Laboratory of Digital Quality Evaluation of Chinese Materia Medica of State Administration of TCM, Guangdong Pharmaceutical University, Guangzhou, China; ^3^ Section of Neuromedical Science, Institute of Natural Medicine, University of Toyama, Toyama, Japan

**Keywords:** Alzheimer’s disease, natural products, neurite outgrowth, neuroprotection, anti-neuroinflammation, brain microenvironment

Alzheimer’s disease (AD) is an aging-related progressing neurodegenerative brain disorder. Extracellular neuritic plaques composed of misfolded amyloid β (Aβ) proteins and intracellular neurofibrillary tangles formed by hyperphosphorylated tau protein are the two typical characteristics of AD. Although AD has been reported for over 100 years, the intrinsic mechanism remains elusive and there are still no well-established clinical strategies to cure or even prevent this disease. Most of the designed molecules targeting Aβ and tau fail to improve cognitive function in clinical trials. Neurite atrophy and synaptic loss underlie the upstream of neuronal death and directly lead to memory impairment in AD (Dickson and Vickers, 2001). Thus, targeting neurite regeneration and synaptic reformation to reconstruct neuronal networks may provide promising therapeutic strategies for AD (Tohda, 2016). This research topic aimed to focus on natural products as novel therapeutics for AD based on neurite regeneration and neuroprotection.

The accumulation of Aβ oligomers promotes axonal transport impairment and follows mitochondrial dysfunction in the distal of neurites, leading to axonal degeneration (Salvadores et al., 2020). In addition, Ooi et al. demonstrated that glyceraldehyde-derived advanced glycation end-products (AGEs), involved in diabetes mellitus-associated AD, triggered tau hyperphosphorylation and abnormal aggregation of toxic AGE-β-tubulin complexes, thus inhibiting neurite outgrowth in human neuroblastoma SH-SY5Y cells. Meanwhile, the AGE inhibitor aminoguanidine and pyridoxamine inhibited the formation of AGE-β-tubulin complexes and mitigated the glyceraldehyde-induced suppression of neurite outgrowth.

Natural products are promising resources for AD intervention, a systematic strategy has been proposed to discover bioactive candidates in natural medicines based on neurite outgrowth, and subsequently to identify target proteins of bioactive compounds by means of drug affinity responsive target stability (DARTS) analysis (Yang et al., 2017). Furthermore, the saponins from *Eleutherococcus senticosus* showed neurite elongation effects in primary cultured cortical neurons and correlated with memory enhancement in normal rats, and 26 saponins were clarified penetrating the blood–brain barrier (Huang et al., 2022). Shi et al. investigated the memory ameliorative effects of baicalein, a well-known flavone, in Aβ42 hippocampal CA1 injected model mice. As a result, baicalein remarkably attenuated Aβ-induced neurite atrophy, spine loss, synaptic dysfunction, and cognitive deficits.

Glial cells play important roles in maintaining brain microenvironmental homeostasis. In response to Aβ stimuli, microglia NLRP3 assembles adaptor protein ASC and procaspase-1 into an inflammasome complex to induce the caspase-1 mediated secretion of IL-1β/IL-18, accompanied by a microglial switch from the protective M2 phenotype to the inflammatory M1 phenotype (Heneka et al., 2013). M1 microglia exerts low expression of degradation enzymes and decreased ability to engulf and degrade Aβ, resulting in increased Aβ deposits. While, naringenin, a natural flavanone, is able to transform the phenotype of M1 microglia to M2 microglia, and increase the expression of Aβ degradation enzymes including neprilysin and insulin degradation enzyme (Yang et al., 2019). The secreted IL-1β from M1 microglia triggers neuronal tau hyperphosphorylation *via* enhancing CaMKII-α and inhibiting PP2A activity (Ising et al., 2019). However, M2 microglia secreted IL-4 inhibits the phosphorylation of tau, thereafter, creating a beneficial microenvironment for the maintenance of regenerated axons. Tang et al. reported that a Chinese medicine formula 9002A ameliorated the increased expression of APP and memory deficits in an Aβ_1-42_ brain-injected mouse model. Sohn et al. investigated a food and medicinal plant, *Ficus erecta*, which could inhibit the activation of microglia and IL-1β production in icv Aβ_1-42_ injected mice. Furthermore, Aβ_1-42_-induced Aβ deposits, neuronal death, and cognitive impairment were also alleviated by *Ficus erecta* ethanol extracts treatment. In addition, Han et al. summarized the lignans, especially dibenzocyclooctene lignans, as potential anti-AD candidates for extensive pharmacological effects such as anti-neuroinflammation, neurite regeneration, and neuro-protection.

**FIGURE 1:**
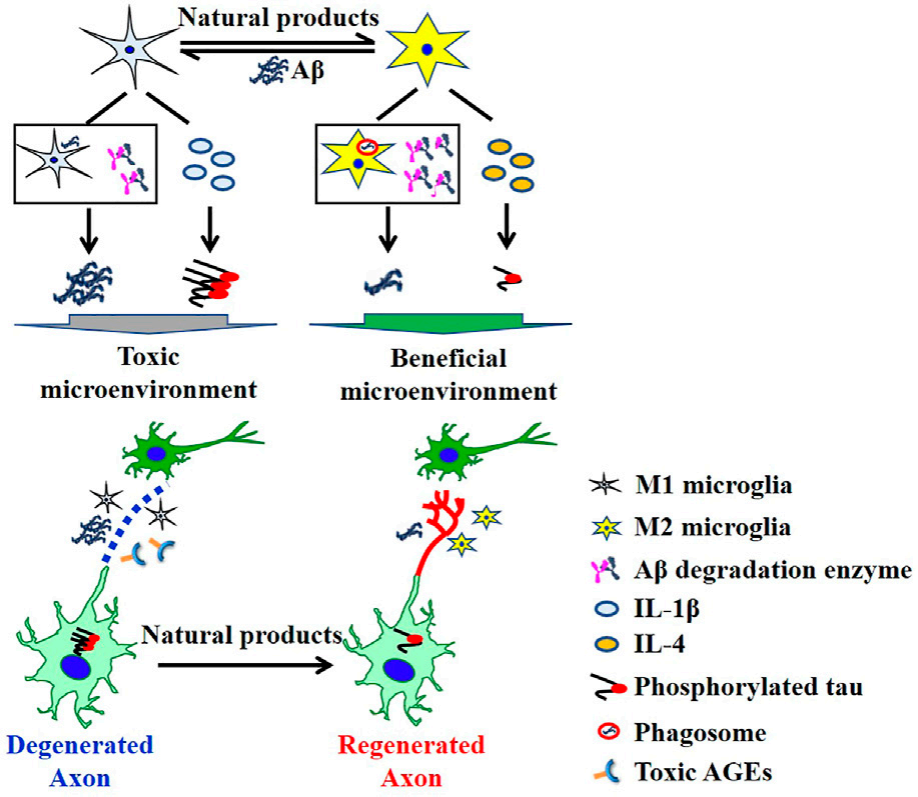
Neurite regenerative strategy as potential AD therapy.

Collectively, neurite regeneration is essential for memory reconstruction, while modulating a beneficial microenvironment by removing toxic AGEs, Aβ plaques, phosphorylated tau protein, and inflammatory cytokines is required for the maintenance of regenerated neurites (Figure 1). Thus, muti-targeting therapies hold great potential to treat AD and traditional medicine-derived natural products precisely correspond to this characteristic.
